# Magnesium Ion Gated Ion Rejection through Carboxylated Graphene Oxide Nanopore: A Theoretical Study

**DOI:** 10.3390/molecules29040827

**Published:** 2024-02-12

**Authors:** Jianjun Jiang, Yusong Tu, Zonglin Gu

**Affiliations:** 1 College of Chemistry and Chemical Engineering, Yangzhou University, Yangzhou 225002, China; jianjunjiang@126.com; 2 Department of Physics, Sanjiang College, Nanjing 210012, China; 3 College of Physical Science and Technology, Yangzhou University, Yangzhou 225009, China

**Keywords:** graphene oxide membrane, molecular dynamics simulation, water desalination

## Abstract

While nanoporous graphene oxide (GO) is recognized as one of the most promising reverse osmosis desalination membranes, limited attention has been paid to controlling desalination performance through the large GO pores, primarily due to significant ion leakage resulting in the suboptimal performance of these pores. In this study, we employed a molecular dynamics simulation approach to demonstrate that Mg^2+^ ions, adhered to carboxylated GO nanopores, can function as gates, regulating the transport of ions (Na^+^ and Cl^−^) through the porous GO membrane. Specifically, the presence of divalent cations near a nanopore reduces the concentration of salt ions in the vicinity of the pore and prolongs their permeation time across the pore. This subsequently leads to a notable enhancement in salt rejection rates. Additionally, the ion rejection rate increases with more adsorbed Mg^2+^ ions. However, the presence of the adsorbed Mg^2+^ ions compromises water transport. Here, we also elucidate the impact of graphene oxidation degree on desalination. Furthermore, we design an optimal combination of adsorbed Mg^2+^ ion quantity and oxidation degree to achieve high water flux and salt rejection rates. This work provides valuable insights for developing new nanoporous graphene oxide membranes for controlled water desalination.

## 1. Introduction

Freshwater scarcity has become a widespread issue due to rapid urbanization and industrialization [1,2,3]. The ever-increasing demand for freshwater necessitates the cost-effective purification of seawater, which constitutes 97.5% of the Earth’s water [4,5]. Reverse osmosis (RO) has been proven to be an efficient method for seawater desalination, characterized by lower energy consumption and capital costs [1,6,7,8]. Currently, RO accounts for 70% of the global seawater desalination capacity [9]. Membrane thickness is a critical factor influencing the quality of the RO water desalination process [4,10]. Traditional RO membranes have limited water flux, making freshwater production energy-intensive. This is mainly because commercial RO membranes require adequate crosslinking to achieve high salt rejection rates, often resulting in relatively thick membranes [11,12,13,14]. In the actual desalination process, using membranes with excellent selectivity and effective pre-treatment and post-treatment processes is crucial for improving RO water quality [15,16]. Nanoporous graphene membranes have garnered significant interest in the development of nanostructured materials [10,17,18,19,20,21,22,23,24,25,26,27,28,29,30]. It has been demonstrated that nanoporous graphene can function as a synthetic water channel, holding great promise for water desalination due to its remarkable properties. These properties include its high aspect ratio and meager transport resistance, attributed to its monoatomic thickness [31].

Prior research has shown that the pore diameters of nanoporous graphene membranes can influence the transport of substances during the water desalination process [10,32]. It has been observed that a critical pore diameter exists below which the permeation of salt ions is effectively blocked [10,32]. While increasing pore size enhances water flux, it simultaneously reduces salt rejection. In practical applications, most synthesized porous graphene membranes possess pore sizes larger than this critical threshold [27,33]. Consequently, researchers often modify the functionalization of the pore periphery or introduce charges to the graphene surfaces to enhance purification efficiency and ion selectivity [4,24,27,30,34,35,36,37,38]. For example, ionizing the functionalization groups on the nanopores’ perimeters can increase water flux at the expense of reduced salt rejection [35]. Utilizing fluorine functionalization has proven effective in preventing the passage of Cl^−^ ions through the membrane, leading to higher salt rejection rates [38]. Due to various interactions such as electrostatic and cation–π interactions [39], divalent cations can be absorbed onto the surfaces of graphene membranes or graphene oxide (GO) membranes, which are synthesized by oxidizing pure graphene membranes. In reference [40], Chen demonstrated precise control of the interlayer spacing of GO membranes through cationic manipulation. It has been established that GO membranes with surface cation adsorption can be effectively employed for water desalination. In this study, we designed a GO membrane with divalent cations (Mg^2+^) adsorbed on the rim of a large pore. Without adsorbed Mg^2+^ ions, the GO membrane with a large pore exhibited high water permeability but low salt rejection, rendering it unsuitable for water desalination. Our results indicate that the nanoporous graphene membrane with adsorbed Mg^2+^ ions acts as a gate, effectively controlling the passage of salt ions, including Na^+^ ions and Cl⁻ ions, across the pore. As a result, such a membrane demonstrates significantly improved salt rejection and meets the requirements for desalination. Moreover, the water permeability of the large pore with adsorbed Mg^2+^ ions remains much higher than that of many other nanopores.

Pore diameter, temperature, applied pressure, and the oxidation degree of the GO membrane are three significant factors influencing water desalination performance. While extensive research has been conducted on the roles of pore diameter, temperature, and applied pressure in water desalination [4,26,33,38], there has been a relative lack of detailed investigation into the impact of the oxidation degree. In this study, we focused on elucidating the influence of the oxidation degree on the transport of water molecules and salt ions through the pore. Our results emphasize the importance of maintaining an appropriate oxidation degree to achieve high water flux and salt rejection rates. Furthermore, we compared the water desalination performance between pores with and without an Mg^2+^ cation, revealing that its presence makes the membrane more robust against changes in oxidation degree.

Recently, molecular dynamics (MD) simulations have proven to be effective in exploring the microscopic desalination mechanism of nanoporous GO membranes, providing insights and details that are not easily attainable through experimental means. In this study, we employed MD simulations to investigate the effect of Mg^2+^ cations adsorbed at the pore’s edge on the desalination behavior of the pore. 

## 2. Results and Discussion

### 2.1. Salt Rejection Rate

In this section, we begin by using a GO membrane with an oxidation degree of 7% as a representative case to elucidate how the pore with adsorbed Mg^2+^ ions regulates the passage of salt ions. One of the critical factors in water desalination is the membrane’s capability to reject ions as the primary objective of water desalination is the separation of ions from saline water. We quantify the salt rejection rate as follows to assess the membrane’s performance in allowing ions to transport through the gated pore [10].

*SR_Na_*_(*Cl*)_ = (1 − *n_Na_*_(*Cl*)_/*N_Na_*_(*Cl*)_) 
*SR_NaCl_* = (1 − *n_NaCl_*/*N_NaCl_*) (1)

**Figure 1:**
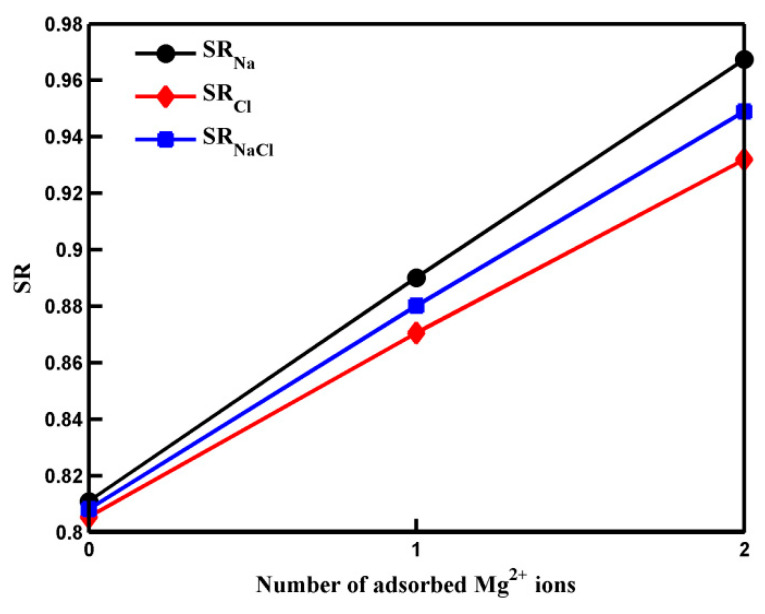
The salt rejection rates of pores with different numbers of adsorbed Mg^2+^ ions. The solid lines are a guide to the eye.

In the above equation, *N_Na_*_(*Cl*)_ and *N_NaCl_* represent the initial numbers of Na^+^ (Cl^−^) ions and total salt ions (the sum of Na^+^ ions and Cl^−^ ions) in the saltwater side at *t* = 0 while *n_Na_*_(*Cl*)_ and *n_NaCl_* denote the numbers of Na^+^ (Cl^−^) ions and total salt ions that have passed through the pore at *t* = *t*_1/2_ (defined as the time when half of the water molecules in the saltwater side have transported through the pore [10]). Figure 1 illustrates the salt rejection rates of the GO membrane with pores adsorbed by zero, one, and two Mg^2+^ ions. Notably, in the absence of the Mg^2+^ ion, the salt rejection rate is statistically equal to the Cl^−^ ion salt rejection rate. However, in the presence of Mg^2+^ cation, the Na^+^ rejection rate exceeds the Cl^−^ rejection rate. This difference arises primarily from the repulsion of Na^+^ ions from the divalent cations. The discrepancy between the two salt rejection rates becomes more pronounced as the number of adsorbed Mg^2+^ ions increases. Both the Cl^−^ ion salt rejection rate and the Na^+^ ion salt rejection rate increase when more Mg^2+^ ions are adsorbed by carboxyl groups. Consequently, the overall salt rejection rate follows the same trend.

**Figure 2:**
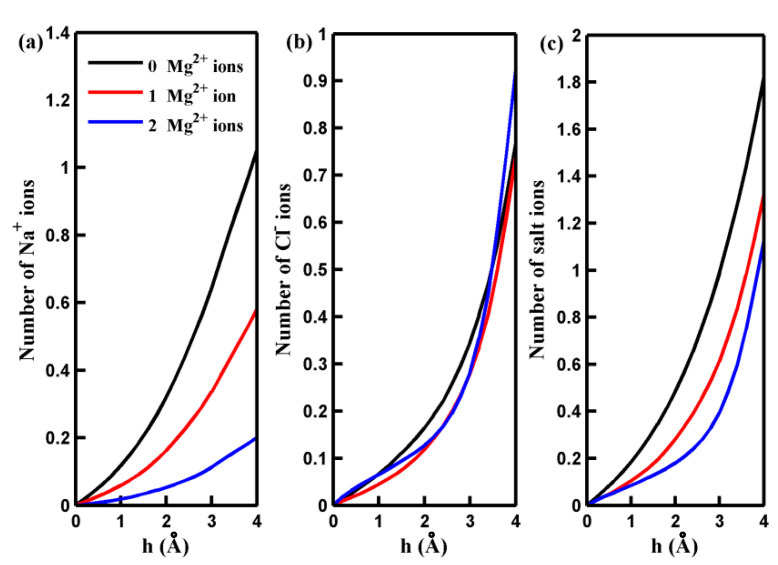
The number of the Na^+^ ions (**a**), Cl^−^ ions (**b**), and salt ions (**c**) accumulating in a cylindrical region below and around the centers of the pores with different numbers of adsorbed Mg^2+^ ions as a function of the height of the cylinder h.

**Figure 3:**
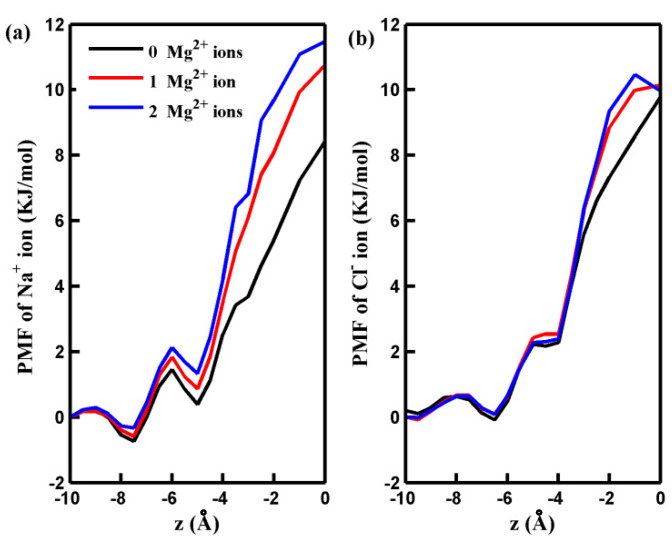
The PMF of Na^+^ ion (**a**) and Cl^−^ ion (**b**) along the *z*-axes of GO membranes with different numbers of adsorbed Mg^2+^ ions. The pore is located at *z* = 0.

To illustrate the observed behavior more clearly, we conducted non-equilibrium MD simulations to analyze the distribution and dynamics of salt ions around the center of the pore. Figure 2 presents the results, showing the number of salt ions accumulating in a cylindrical region (more illustration below) as a function of the cylinder height (h). As depicted in Figure 2, an increase in the number of adsorbed Mg^2+^ ions decreases the quantity of the Na^+^ ions around the pore. This decrease can be attributed to the electrostatic repulsion force between the adsorbed Mg^2+^ ions and Na^+^ ions. While the adsorbed Mg^2+^ ions exert an attractive force on Cl^−^ ions, the quantity of Cl^−^ ions near the pore displays a slightly nonmonotonic variation with an increased number of adsorbed Mg^2+^ ions. This phenomenon is partly because the attractive force acting on Cl^−^ ions through the Na⁺ ions gathering near the pore decreases as the number of adsorbed Mg^2+^ ion ions rises. For a more in-depth investigation into the mechanisms underlying the distribution of salt ions around the pore and their passage through it at the molecular level, we calculated the potential of mean force (PMF) of the ions along the *z*-axis of the simulation box. These calculations were performed under an applied pressure of 100 MPa, and the results are depicted in Figure 3. The PMF profiles indicate that the permeation of Na^+^ and Cl^−^ ions across the pore is energetically unfavorable, making the spontaneous passage of Na^+^ and Cl^−^ ions impossible. When Mg^2+^ ions are adsorbed on the rim of the pore, the free energy barrier for Na^+^ ions to be transported through the pore increases significantly. This accounts for the observed decrease in the number of Na^+^ ions around the pore in the presence of adsorbed Mg^2+^ cations. Notably, the free energy barrier for Cl^−^ ions remains nearly unchanged in the presence of Mg^2+^. The behavior of the PMF profile for Cl^−^ ions clarifies that the adsorption of Mg^2+^ ions on the pore does not result in the aggregation of Cl^−^ ions inside the pore.

In summary, the analysis of salt ion distribution near the pore and the PMF profiles strongly suggest that the Mg^2+^ cation adsorbed on the edge of the pore impedes the passage of Na^+^ ions. Consequently, the Na^+^ ion salt rejection rate increases. However, the slight change in Cl^−^ ion concentration near the pore is not the primary cause of the increase in the Cl^−^ ion salt rejection rate. To further investigate the influence of the dynamics of salt ions on their transport through the pore, we conducted non-equilibrium MD simulations. The dynamics of salt ions were examined by calculating the permeation time autocorrelation function *C*(*t*), defined as follows.

*C_Na_*_(*Cl*)_(*t*) = 〈*H_Na_*_(*Cl*)_(*t*)*H_Na_*_(*Cl*)_(0)〉/〈*H_Na_*_(*Cl*)_(0)^2^〉 *C_NaCl_*(*t*) = 〈*H_NaCl_*(*t*)*H_NaCl_*(0)〉/〈*H_NaCl_*(0)^2^〉 (2)

**Figure 4:**
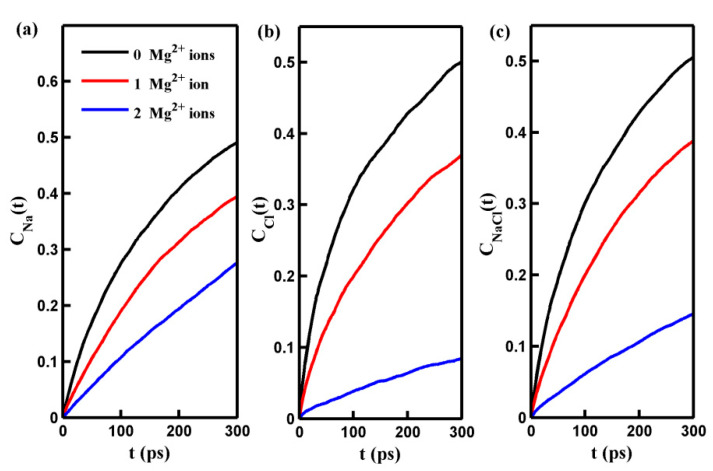
The permeation time autocorrelation function of Na^+^ ion (*C_Na_*(*t*)) (**a**), Cl^−^ ion (*C_Cl_*(*t*)) (**b**), and salt ion (*C_NaCl_*(*t*)) (**c**) in pores with different numbers of adsorbed Mg^2+^ ions as a function of time *t*.

Here, *C_Na_*_(*Cl*)_(*t*) and *C_NaCl_*(*t*) represent the permeation time autocorrelation function of Na⁺ (Cl^−^) ions and total salt ions, and *H_Na_*_(*Cl*)_(*t*) (*H_NaCl_*(*t*)) is a binary function that equals 1 if one ion is initially located inside the pore at *t* = 0 and continuously exists above the center of the pore at any time greater than *t* and equals 0 otherwise. A water molecule or ion within a cylindrical region with a diameter of 1.6 nm and a height of 0.2 nm below and around the center of the pore is considered to be within the pore [30], also demonstrated below. This permeation time autocorrelation function (Equation (2)) is a suitable measure of the permeation probability of ions or water molecules passing through the pore. The results of the permeation time autocorrelation function *C*(*t*) are presented in Figure 4. The figure shows that the rate of increase in *C*(*t*) for ions decreases over simulation time. This phenomenon arises because, as shown in Table 1, ions have finite residence times inside the pore. Furthermore, the residence time of ions increases with an increasing number of adsorbed Mg^2+^ cations. Therefore, as the number of adsorbed cations increases, the decrease in the rate of *C*(*t*) with time becomes less pronounced. The evolution of *C*(*t*) with time suggests that estimating the permeation probability of ions using *C*(*t*) with small time intervals (*t*) is more appropriate. Figure 4b demonstrates that the adsorption of Mg^2+^ ions on the pore significantly hinders the mobility of Cl^−^ ions near the pore center and prolongs their permeation time through the pore. This dynamic behavior of Cl^−^ ions helps explain why an increase in the number of adsorbed Mg^2+^ cations contributes to the enhancement of the Cl^−^ ion salt rejection rate. It should also be noted that the permeation probability of Na⁺ ions, similar to Cl^−^ ions, decreases with increasing adsorbed Mg^2+^ cation quantity. Extending the permeation time for Na^+^ ions improves the Na^+^ ion salt rejection rate. The results presented in this section demonstrate that the system can function as a gated system for controlling and regulating the passage of salt ions through the pore.

### 2.2. Water Flux

**Figure 5:**
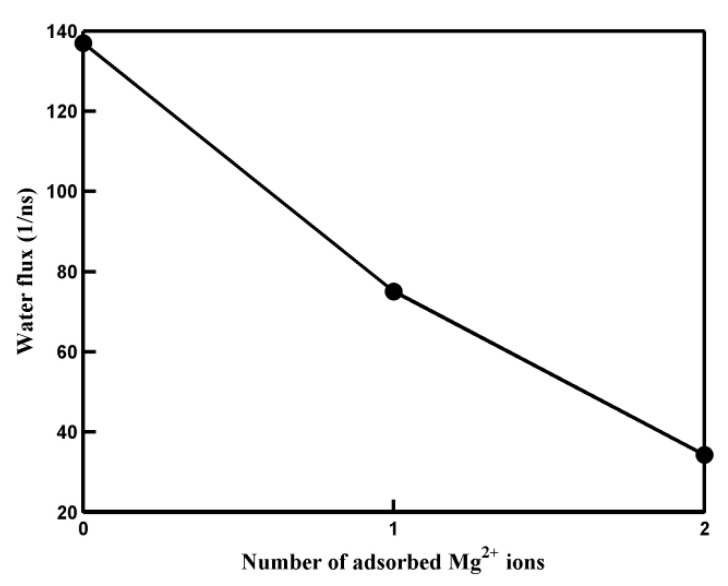
The water flux of pores with different numbers of adsorbed Mg^2+^ ions. The solid line is a guide to the eye.

Like the salt rejection rate, water flux is another crucial factor in water desalination using membrane materials. The water flux was measured by counting the net number of water molecules passing through the pore during the course of the simulation time [41]. Figure 5 illustrates the results for water flux as a function of the number of adsorbed Mg^2+^ cations. It is observed that the water flux decreases as the number of adsorbed Mg^2+^ cations increases. Nevertheless, as indicated in Table 2, even in the case of a pore with two adsorbed Mg^2+^ cations, its water permeability still reaches an impressive value of 58.7 L/cm^2^/day/MPa. This level of water permeability significantly surpasses that of many other nanopores, including MoS_2_ nanopores, hydrogenated graphene nanopores, phosphorene, and g-C_3_N_4_ nanopores. Furthermore, the salt rejection of the pore with two adsorbed Mg^2+^ cations meets the requirements for desalination purposes. As explained in Supplementary Materials Section S2, the variation in water flux is attributed to the fact that the adsorption of Mg^2+^ ions on the pore’s rim can influence the permeation probability of water molecules passing through the pore.

### 2.3. Effects of the Oxidization Degree on Desalination

**Figure 6:**
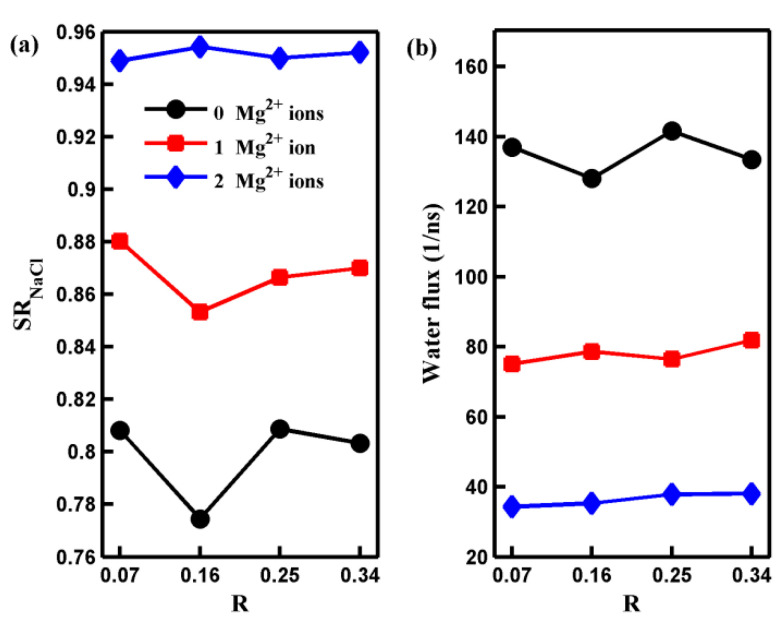
The salt rejection rate (**a**) and the water flux (**b**) of the nanoporous GO membranes with different numbers of adsorbed Mg^2+^ ions and oxidization degree R. The solid lines are a guide to the eye.

**Figure 7:**
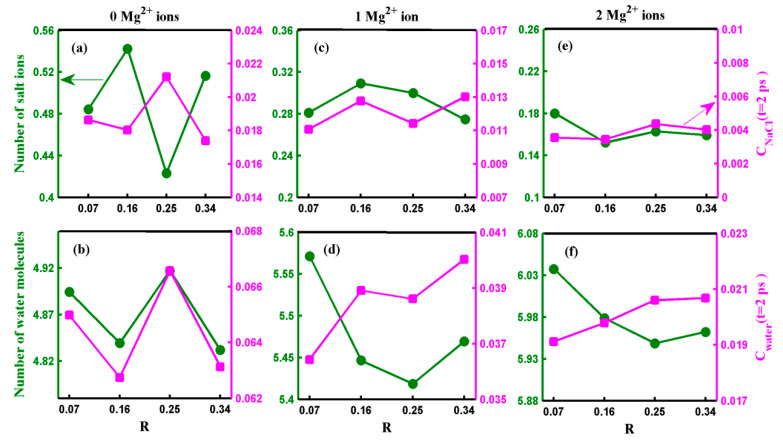
(**a**–**f**) show the number of the salt ions (water molecules) located inside the pore and the salt ion (water molecule) permeation time autocorrelation function at time *t* = 2 ps for each oxidization degree R. The figures along the parallel direction represent the pores with 0, 1, and 2 adsorbed Mg^2+^ ions. The solid lines are a guide to the eye.

Now, let us discuss the influence of the oxidization degree on the transport of water molecules and salt ions through the pore. Figure 6 presents the salt rejection rate and water flux of the GO membrane with various numbers of Mg^2+^ ions as a function of the oxidization degree. As depicted in Figure 6, neither the salt rejection rate nor water flux exhibits a monotonic variation with oxidization degree. Furthermore, the variation in the salt rejection rate or water flux becomes smaller as the number of adsorbed Mg^2+^ cations increases. These results reveal that when the oxidization degree changes, the pore with adsorbed Mg^2+^ cations exhibits a more robust water desalination performance than those without adsorbed Mg^2+^ cations. To understand the mechanism behind the effect of oxidization degree on water desalination performance, we provide the results for all systems regarding the number of salt ions (water molecules) located inside the pore and the ion (water molecule) permeation time autocorrelation function *C*(*t* = 2 ps) in Figure 7.

In the case of the pore without an Mg^2+^ cation, the salt rejection rate and water flux exhibited peaks and troughs at R = 0.25 and R = 0.16, respectively, as shown in Figure 6. The result displayed in Figure 7a reveals that the number of salt ions inside the pore without an Mg^2+^ cation changes significantly but not monotonically with the oxidization degree. In the case of R = 0.25 (R = 0.16), although the permeation probability of salt ions is the highest (the second smallest) among the four cases, the number of ions inside the pore is the smallest (largest). Thus, the substantial difference in the number of ions inside the pore between R = 0.25 and R = 0.16 leads to an extensive range of variation in the salt rejection rate without adsorbed Mg^2+^ cations. As demonstrated in Figure 7b, the distribution of water inside the pore is nearly the smallest (the largest) among all the systems when R = 0.16 (R = 0.25). These results suggest the presence of competitive penetration between salt ions and water molecules [32]. Although the number of water molecules in the pore is not greatly affected by changes in the oxidization degree, the water flux of the pore without adsorbed Mg^2+^ cations exhibits a significant change with the oxidization degree. This is mainly because the variation trend of the permeation probability of water with respect to the oxidization degree is similar to the variation trend of the number of water molecules inside the pore as the oxidization degree changes. Therefore, both the permeation time of water molecules and the number of water molecules within the pore play crucial roles in water desalination. However, the situation is somewhat different when considering the salt rejection rate of the pore with adsorbed Mg^2+^ cations. The salt rejection rate of the pore with adsorbed cations is primarily influenced by two significant factors: the number of salt ions inside the pore and the permeation time of the salt ions. However, since the oxidization degree of the GO membrane has minimal impact on the number of salt ions inside the pore and the permeation probability of ions, the salt rejection rate of the pore with adsorbed Mg^2+^ cations fluctuates within a narrow range. On the other hand, the water flux of the pore with adsorbed Mg^2+^ cations is co-determined by the permeation time of water molecules across the pore and the number of water molecules inside the pore, similar to the case of the pore without adsorbed Mg^2+^ cations. However, due to the pronounced difference between the change in the number of water molecules inside the pore and the permeation probability of water molecules with respect to the oxidization degree, the small variations in either the number of water molecules inside the pore or the permeation probability of water molecules alone cannot result in a significant change in the water flux.

### 2.4. Water Desalination Performance of Another Porous GO Membrane

The pore diameter is a crucial factor in determining the separation performance of the GO membrane. To investigate the role of the adsorbed divalent cation on the water desalination performance of the GO membrane with a large pore diameter of 2.15 nm (as shown in Figure S6), we conducted additional non-equilibrium MD simulations. The results indicate that the influence of the adsorbed Mg^2+^ cation on the water desalination performance of the pore with a diameter of 2.15 nm is similar to that of the adsorbed Mg^2+^ cation observed for the pore with a diameter of 1.29 nm. Further details can be found in Supplementary Materials Section S3 of the paper.

## 3. Materials and Methods

**Figure 8:**
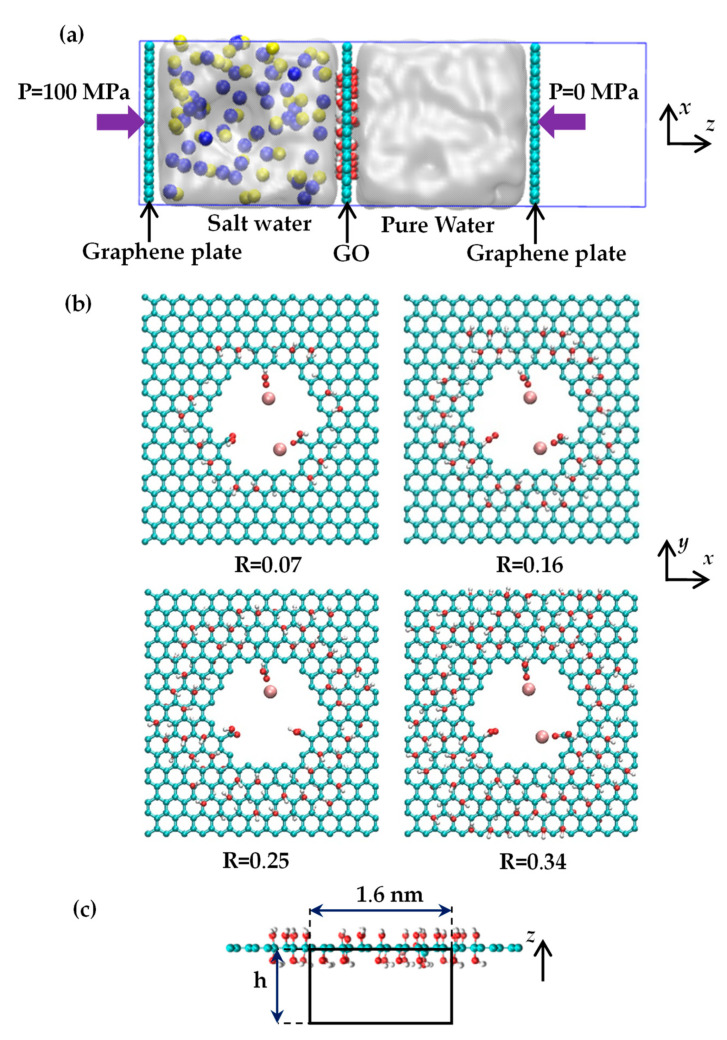
(**a**) Side view of the gated system for investigating desalination through the GO membrane. The yellow and blue balls denote the sodium and chloride ions. (**b**) Atomistic structure of the nanoporous GO membrane with a variation in oxygen degree. One or two Mg^2+^ ions are placed near the carboxylic oxygen. The red, cyan, white, and pink spheres represent the oxygen, carbon, hydrogen, and magnesium atoms, respectively. (**c**) The black box in the figure denotes a cutout view of a cylindrical region with a diameter of 1.6 nm and a height of h below and around the center of the pore in the GO membrane.

Figure 8a illustrates the gated system used in our study. This system consists of two slabs: one containing a saltwater (NaCl solution) region and the other containing pure water, separated by a nanoporous GO membrane at *z* = 0. The two graphene plates are free to move in the z-direction to control the system’s pressure. The simulation model’s dimensions are 3.30 × 3.52 × 10.50 nm in the *x*, *y*, and *z* directions, respectively, with periodic boundary conditions applied in all directions. Without Mg^2+^ cations, the saltwater portion contains 46 Na^+^ ions, 46 Cl^−^ ions, and 1126 water molecules, resulting in a salt concentration of 132.8 g/L. This elevated salinity level is advantageous for obtaining precise statistical results regarding ion permeation through the pore [10,30]. The pure water section comprises 1120 water molecules. 

Given that GO layers predominantly consist of hydroxyl groups [30,44], the GO membrane utilized in our study features hydroxyl groups on both sides of the graphene basal plane. Figure 8b shows the placement of a single nanopore at the center of the GO membrane. The pore diameter, calculated using the formula $d=2\sqrt{A/\pi \;}$, measures 1.29 nm [10]. This pore size is sufficiently large to facilitate the transport of salt ions from saltwater to pure water and can be readily created experimentally [27]. Three carboxyl groups are also attached to the carbon atoms on the pore’s edge. As oxidized regions tend to form a continuous network across the GO membrane and consistently coexist with nanopores in the same area, the hydroxyl groups are distributed in a circular region surrounding the pore [32]. The oxidation degree of the GO membrane is characterized by the R = *n_oh_*/*n_c_* ratio, which ranges from 7% to 34%, where *n_oh_* represents the number of hydroxyl groups and *n_c_* denotes the number of carbon atoms. Figure 8b provides top views of the GO membranes. The figure illustrates that the Mg^2+^ ions were initially positioned near the carboxylic oxygen (double-bonded) sites. Due to the strong electrostatic interaction between Mg^2+^ ions and carboxylic oxygen, the Mg^2+^ ions consistently remain near the carboxylic oxygen throughout all MD simulations (for further details, please refer to Supplementary Materials Section S1). Figure 8b shows that the number of Mg^2+^ ions adsorbed to the carboxyl groups varies between 1 and 2. Except the counter ions of Na^+^, there are another 2 or 4 Cl^−^ ions that should be added to maintain the electroneutrality of the system with Mg^2+^ ions.

All MD simulations were conducted using the Gromacs simulation package in the canonical (NVT) ensemble at a temperature of 300 K, employing a velocity-rescale thermostat for temperature control [45]. The GROMOS53a6 force field was utilized to describe both the GO membrane and the ions [46,47] while water molecules were represented using the standard SPC model [48]. The LINCS algorithm was employed to constrain all bonds [49], and the classical equations of motion were integrated using the leapfrog algorithm with a time step of 1 fs [50]. The particle mesh Ewald method and Lennard–Jones potential were implemented to calculate electrostatic interactions and non-bonded interactions between different particles [51,52]. The cutoff for Lennard–Jones or electrostatic interactions was set at 1.2 nm. All atoms in the GO membrane, except those belonging to oxidation functional groups, were fixed throughout all simulations to mitigate the impact of mechanical deformation phenomena on saltwater permeation behavior [10].

Initially, we equilibrated the systems at 300 K while keeping water molecules near the center of the GO membrane fixed to separate the saltwater and pure water regions. The pressures on the two graphene plates were maintained at zero during this process. Following equilibration for 1 ns, as illustrated in Figure 8a, we applied pressures of 100 MPa and 0 MPa to the left and right graphene plates, respectively to conduct non-equilibrium MD simulations. Such a significant pressure difference ensures that accurate data for water flux and salt rejection are acquired within a finite simulation time accessible through MD simulations [10,26]. The pressure applied to the graphene plate can be calculated using the moving acceleration rate of the plates as follows [32]:

*P* = *Nma*/*S* (3)

Here, *N* is the total number of carbon atoms in the plate, *m* is the mass of a carbon atom, *S* is the cross-sectional area, and *P* is the applied pressure. Throughout the non-equilibrium simulation, coordinates were saved every 2 ps. To obtain converged quantities for each system, such as salt rejection and water flux, 10–20 non-equilibrium runs of 5–20 ns were performed with independent initial configurations.

The PMF was used to provide a semi-quantitative understanding of the energetics for ion permeation through the pore [30,38]. The PMF was calculated through 10–20 non-equilibrium simulations. The adaptive biasing forcing algorithm was employed to determine the PMF as usual [53,54,55]. The reaction coordinate for the GO membrane was set in the z-direction, ranging from −10 Å to 0 Å (center of the membrane pore), preceding the pore.

## 4. Conclusions

In conclusion, our molecular dynamics simulations demonstrate that the nanoporous GO membrane adsorbing the Mg^2+^ cation onto the rim of the pore can effectively act as a gate to control the passage of salt ions across the large pore. This significantly improves the desalination performance of the nanoporous GO membrane by adsorbing Mg^2+^ cations. The simulation results reveal that the transport of salt ions (and water molecules) through the pore is closely correlated with both the distribution of salt ions (and water molecules) near the center of the pore as well as the salt ion (water molecule) permeation time across the pore. The Mg^2+^ cations absorbed on the pore reduce the concentration of salt ions around the center of the pore due to strong repulsive forces between the Na^+^ ions and the Mg^2+^ cations. Moreover, the increase in the number of adsorbed Mg^2+^ cations increases the time it takes for salt ions to permeate through the pore, thereby improving the salt rejection rate. However, the Mg^2+^-adsorbed pore compromises the passage of water molecules through the pore. As the divalent cation can be adsorbed on the surface of the GO membrane very stably owing to their interactions (e.g., the density functional theory (DFT) calculation results in Supplementary Materials Section S1 disclose that there is strong interaction between the Mg^2+^ cation and the nanoporous GO membrane), one possible way to obtain the nanoporous GO membrane with the adsorption of Mg^2+^ cations is to soak the freestanding nanoporous GO membrane in magnesium-ion water solution for some time during the membrane fabrication process, similar to previous research [40].

The role of the oxidation degree on the desalination performance of the nanoporous GO membrane was also investigated. The mechanisms underlying the variation in the salt rejection rate and water flux with the oxidation degree were elucidated. The number of water molecules (ions) near the center of the pore and the permeation time of water molecules (ions) across the pore are the two key factors influencing water flux (and salt rejection rate) as the oxidation degree of GO varies. The simulations reveal the presence of competitive penetration between salt ions and water molecules in the system, which can be beneficial for the nanoporous GO membrane to achieve high water flux and a perfect salt rejection rate at certain oxidation degrees. Our work suggests that adsorbed Mg^2+^ cations on the nanoporous GO membrane offer a promising avenue for optimizing GO membranes for improved water desalination performance.

## Figures and Tables

**Table 1:** The residence times of ions and water molecules located in the pores with an oxidization degree R = 0.07.

Number of Adsorbed Mg^2+^ Ions	Na^+^ (ps)	Cl^−^ (ps)	Water Molecule (ps)
0 Mg^2+^ ions	10.7316	8.1324	3.6078
1 Mg^2+^ ion	12.2970	8.9998	8.2718
2 Mg^2+^ ions	21.5172	10.9348	12.4690

**Table 2:** The comparison of the desalination performances of the pore and other RO membranes.

RO Membrane	Water Permeability (L/cm^2^/Day/MPa)	Salt Rejection (%)
MoS_2_ nanopore [11]	16	100
Hydrogenated graphene nanopore [10]	39	100
Phosphoerne nanopore [42]	11	100
g-C_3_N_4_ nanopore [43]	15	100
Hydroxylated graphene nanopore [10]	66	100
Pore without adsorbed Mg^2+^ ions (current job)	234	81
Pore with one adsorbed Mg^2+^ ion (current job)	128	88
Pore with two adsorbed Mg^2+^ ions (current job)	59	95

## Data Availability

The raw data supporting the conclusions of this article will be made available by the authors on request.

## Supplementary Materials

The following supporting information can be downloaded at: https://www.mdpi.com/article/10.3390/molecules29040827/s1, Figure S1: The distance between the Mg^2+^ ion and the carboxylic oxygen of the system; Figure S2: The configuration of the graphene sheet with one pore and one adsorbed magnesium ion [56,57,58]; Figure S3: The number of water molecules accumulating in a cylindrical region; Figure S4: The PMF of a water molecule along the z-axis of GO membranes with different numbers of adsorbed Mg^2+^ ions; Figure S5: The permeation time autocorrelation function of water molecules in pores with different numbers of adsorbed Mg^2+^ ions; Figure S6: Atomistic structure of the GO membrane; Figure S7: The salt rejection rate and the water flux of the GO membranes with a large pore diameter of 2.15 nm vs. the oxidization degree R.
