# Supplementary material for: Magnesium Ion Gated Ion Rejection through Carboxylated Graphene Oxide Nanopore: A Theoretical Study
Source: Molecules. 2024 Feb 12;29(4):827. doi: 10.3390/molecules29040827 (PMC10892045; doi:10.3390/molecules29040827)
Supplement: Supplementary file 1 [file molecules-29-00827-s001.zip › molecules-2867393-supplementary.pdf]

Supplementary Materials

1. Evidence for the adsorption of magnesium ions on the periphery of the pore

To the Mg^{2+} ions being adsorbed on the rim of the pore, we present the distance between the Mg^{2+} ion and its neighboring carboxylic oxygen in the gating system depicted in Figure 8(b) with one adsorbed Mg^{2+} ion during one non-equilibrium MD simulation process in Figure S1. The results show that the distance between these two atoms fluctuates slightly around the average value of 1.98 Å, indicating that the Mg^{2+} ion remains stable near the carboxylic oxygen throughout the non-equilibrium MD simulation process.

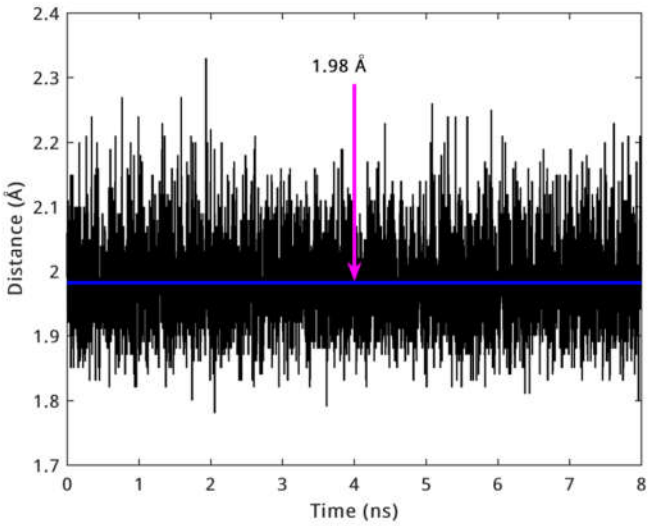

Figure S1. The distance between the Mg^{2+} ion and the carboxylic oxygen of the system shown in Figure 8 with one adsorbed Mg^{2+} ion vs. the simulation time. The horizontal blue line represents the mean values of the distance.

Furthermore, we conducted DFT calculations to determine the binding energies (E_b) of magnesium ions with the graphene pore. The configuration of the graphene sheet with one pore, as obtained from the DFT calculation, is illustrated in Figure S2. The graphene sheet comprises 90 carbon and 29 hydrogen atoms, with one carboxyl group attached to a carbon atom on the pore's edge. Initially, one magnesium ion was positioned near the

carboxyl group. The DFT calculations were carried out using the Gaussian 09 software package [56], with implicit water present. Geometric configurations were optimized using the B3LYP functional and the 6-31G(d) basis set [57]. The binding energy E_b is calculated as follows:

$$E_b = E_{Mg@graphene} - E_{Mg} - E_{graphene}$$

Here, $E_{Mg@graphene}$ represents the energy of the graphene pore with one adsorbed magnesium ion, E_{Mg} is the energy of the magnesium ion, and $E_{graphene}$ is the energy of the graphene pore. The DFT calculation results indicate that E_b has a value of -121.72 kJ/mol, which is approximately six times greater than the hydrogen bond energy in liquid water (approximately 21 kJ/mol [58]). This DFT calculation outcome further confirms that magnesium ions can be stably adsorbed on the rim of the pore.

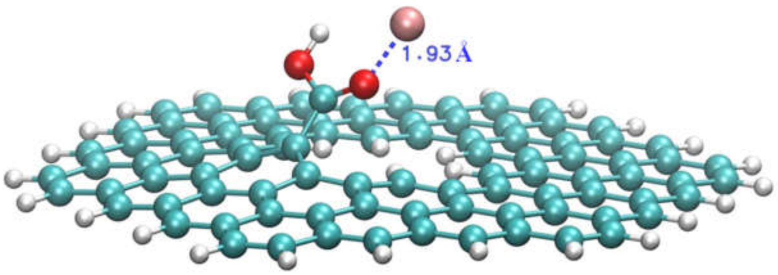

Figure S2. The configuration of the graphene sheet with one pore and one adsorbed magnesium ion. The distance between the magnesium ion and carboxylic oxygen is 1.93 Å.

2. The permeation mechanism of water molecules through the pore

To provide insights into the water permeation mechanism through the pore, we first examine the impact of adsorbed Mg^{2+} cations on the distribution of water molecules around the pore. Figure S3 illustrates the number of water molecules accumulating around the center of the pore. This figure shows that the number of water molecules near the pore increases with adsorbed Mg^{2+} cations. This observation suggests that the strong hydrophilicity of magnesium of Mg^{2+} cation ions contributes to the aggregation of water molecules around the pore. To gain a more detailed understanding of the distribution of

water molecules near the graphene nanopore, we calculated the PMF for a water molecule along the *z*-axis of the simulation box. Figure S4 presents the PMF results for all systems. The PMF curves for all cases exhibit a sharp increase around the center of the pore, indicating that water molecules must overcome an energy barrier to enter the pore. Furthermore, the PMF of a water molecule does not vary significantly along the *z*-axis within a small parameter region (-0.2 nm < *z* < 0 nm), suggesting that a water molecule can be found within the pore in this region. It is also evident from the PMF results that the PMF of a water molecule near the pore becomes lower as the number of adsorbed Mg^{2+} ions increases. Analyzing the behavior of the PMF of a water molecule can help explain why the number of water molecules inside pores with more Mg^{2+} ions is greater than in pores with fewer Mg^{2+} ions. However, to understand the reason for the decrease in water flux, we need to estimate the permeation probability of water molecules in the pore by calculating the permeation time autocorrelation function $C_{water}(t)$, which is defined as

$$C_{water}(t)=\langle H_{water}(t)H_{water}(0)\rangle/\langle H_{water}(0)^2\rangle$$

Here, $H_{water}(t)$ represents a binary function that equals 1 if a water molecule is located inside the pore at t=0 and remains above the center of the pore at any time greater than t, and it equals zero otherwise. The results of the permeation time autocorrelation function for water $C_{water}(t)$ molecules are shown as a function of time t in Figure S5. As the number of adsorbed Mg^{2+} cations increases, the time required for a water molecule to pass through the pore also increases. The reduced permeation probability of water molecules passing through the pore with adsorbed Mg^{2+} ions decreases water flux.

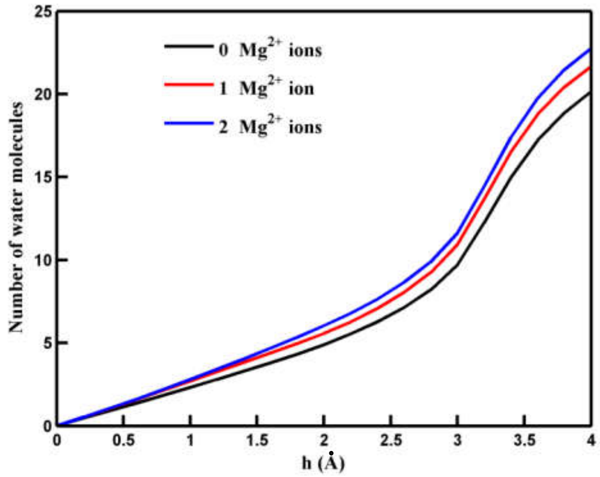

Figure S3. The number of water molecules accumulating in a cylindrical region (as shown in Figure 8(c)) below and around the center of the pores with different numbers of adsorbed Mg^{2+} ions as a function of the height of the cylinder h.

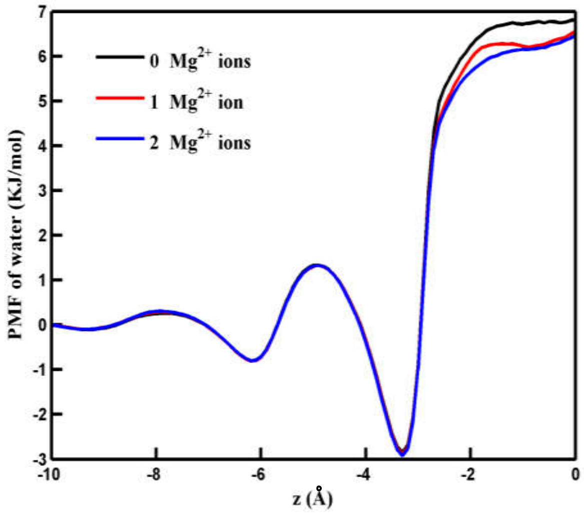

Figure S4. The PMF of a water molecule along the *z*-axis of GO membranes with different numbers of adsorbed Mg^{2+} ions. The pore is located at *z*=0.

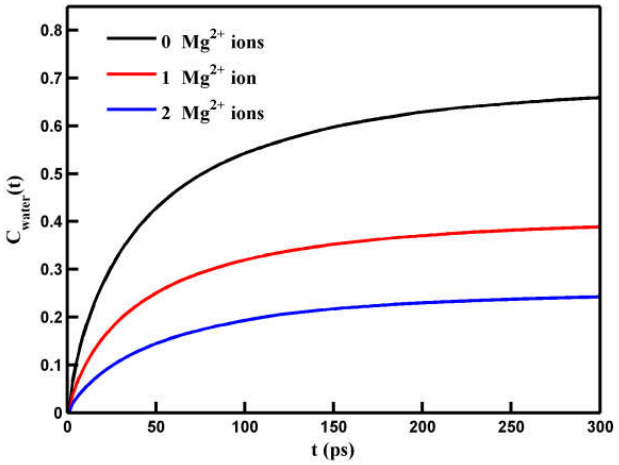

Figure S5. The permeation time autocorrelation function of water molecules in pores with different numbers of adsorbed Mg^{2+} ions as a function of time *t*.

3. The water desalination performance of the large pore with a diameter of 2.15 nm

Figure S6 illustrates the adsorption of 1, 3, or 5 Mg^{2+} ions on the carboxyl groups attached to the rim of a large pore with a diameter of 2.15 nm. Figure S7 shows the results of salt rejection rate and water flux for the large pore with varying numbers of Mg^{2+} ions obtained from non-equilibrium MD simulations at a pressure of 100 MPA. Similar to the behavior observed in the case of the smaller pore with a diameter of 1.29 nm, the adsorbed Mg^{2+} ions on the rim of the larger pore with a diameter of 2.15 nm effectively control the passage of salt ions and water molecules through the pore. As the number of adsorbed Mg^{2+} cations increases, the salt rejection rate improves, while the water flux decreases. Additionally, the presence of adsorbed Mg^{2+} cations helps maintain the robustness of the water desalination performance of the pore when the oxidation degree changes.

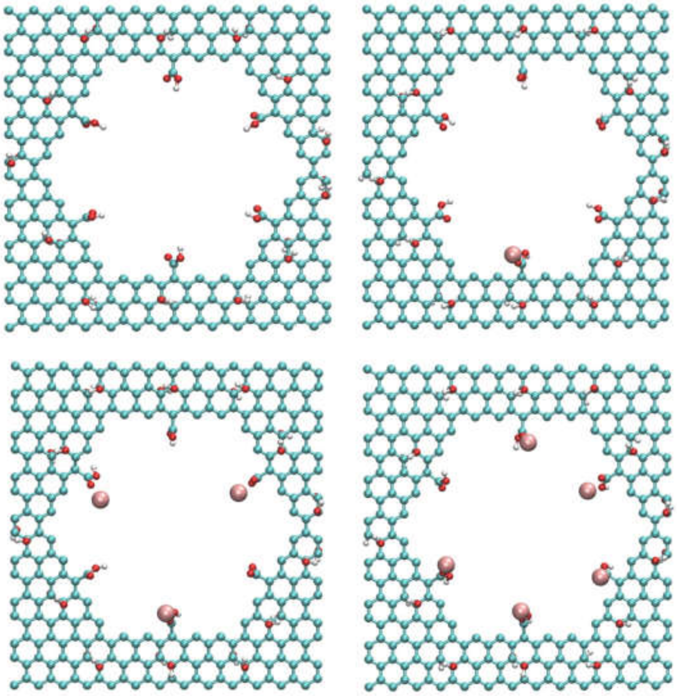

Figure S6. Atomistic structure of the GO membrane with a large pore diameter of 2.15 nm. 0, 1, 3, or 5 Mg^{2+} ions are placed near the carboxylic oxygen.

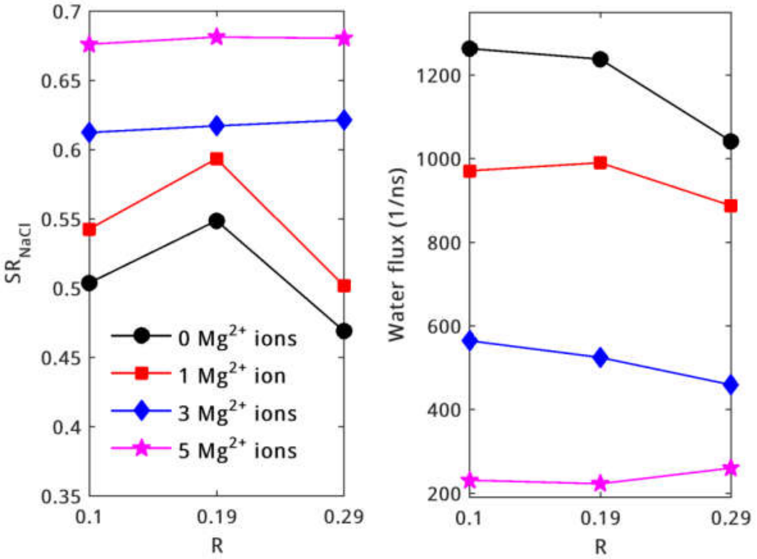

Figure S7. The salt rejection rate and the water flux of the GO membranes with a large pore diameter of 2.15 nm vs. the oxidization degree R. The solid lines are a guide to the eye.
